# Summary of the best evidence for nutritional support programs in nasopharyngeal carcinoma patients undergoing radiotherapy

**DOI:** 10.3389/fnut.2024.1413117

**Published:** 2024-07-31

**Authors:** Xiaomei Fan, Huixia Cui, Shasha Liu

**Affiliations:** ^1^Department of Nursing, Jinzhou Medical University, Jinzhou, China; ^2^First Affiliated Hospital of Chengdu Medical College, Chengdu, China; ^3^School of Nursing, Wannan Medical College, Wuhu, China; ^4^Chengdu BOE Hospital, Chengdu, China

**Keywords:** radiotherapy, nutritional support, evidence-based nursing, nasopharyngeal carcinoma, summary of evidence

## Abstract

**Objective:**

To evaluate and summarize the best evidence for nutritional support in patients receiving radiotherapy for nasopharyngeal carcinoma and to offer guidance for clinical practice.

**Background:**

Patients with nasopharyngeal carcinoma undergoing radiotherapy often experience a high prevalence of malnutrition, and there is a lack of compiled guideline recommendations, which complicates the provision of a reference for clinical nursing.

**Methods:**

A systematic literature search revealed the best evidence of nutritional support for nasopharyngeal carcinoma patients undergoing radiotherapy. Included in the review were various types of literature, such as clinical guidelines, expert consensus, systematic evaluations, meta-analyses, evidence summaries, and original studies. The evidence was graded according to the Australian Joanna Briggs Institute Centre for Evidence-Based Health Care Evidence Pre-Grading System (2016 version). Data were gathered from a range of sources, including BMJ Best Practice, the Scottish Intercollegiate Guidelines Network, the Cochrane Library, Embase, PubMed, Web of Science, CINAL, CNKI, the WanFang database, SinoMed, the Yi Maitong Guidelines Network, Dingxiangyuan, the Chinese Nutrition Society, the European Society for Clinical Nutrition and Metabolism website, and the American Society for Parenteral and Enteral Nutrition website. The search spanned from January 2013 to 2023.

**Results:**

A comprehensive review identified a total of 3,207 articles, comprising six guidelines, eight expert consensus articles, four systematic evaluations, five randomized controlled trials, two cohort trials, and two observational studies. From these articles, we synthesized 63 pieces of evidence spanning six domains: nutritional risk screening and assessment, nutrient requirements, nutritional support, management of nutritional symptoms, functional exercise, and nutritional monitoring and follow-up.

**Conclusion:**

A total of lines of evidence supporting nutritional support for nasopharyngeal carcinoma patients receiving radiotherapy were summarized. However, the evidence should be combined with the actual clinical situation, and it should be validated in the future by combining large-sample and multicenter studies to provide a more scientific and beneficial nutritional support program for nasopharyngeal carcinoma patients receiving radiotherapy.

## 1 Introduction

Nasopharyngeal carcinoma, a malignant tumor arising in the mucosal epithelium of the nasopharynx, demonstrates a notable geographic variation in its occurrence (1). It is more prevalent in East Asia and Southeast Asia, with a higher incidence among males and a peak occurrence between the ages of 40 and 59 (2, 3). This cancer constitutes a common type of malignant head and neck tumor in China.

The primary treatment approach for nasopharyngeal carcinoma involves radiotherapy or a combination of therapies with radiotherapy as the cornerstone (4). Common side effects of radiotherapy for this type of cancer include acute radiation mucositis, radiation dermatitis, radiation-induced damage to salivary glands, and bone marrow suppression. These side effects often manifest as alterations in taste, oropharyngeal and throat pain, dry mouth, accumulation of mucus in the oropharynx and throat, difficulty in chewing or swallowing, and pain, which can impede the intake or absorption of nutrients (5, 6). Malnutrition is a frequent complication in patients undergoing radiotherapy for nasopharyngeal carcinoma, typically occurring early in treatment and worsening as treatment progresses (7, 8). Zhuang et al. (9) found that 69.0% of patients experienced malnutrition by the end of radiotherapy, while Wei et al. (10) reported a severe malnutrition incidence rate of 80.7% during radiotherapy. Hong et al. (11) observed that 20.19% of patients experienced more than a 10% weight loss by the end of radiotherapy. Additionally, a study (12) showed that the prevalence of malnutrition increased from 16.8% before treatment to 91.2% by the end of treatment. Nutritional support is crucial in the clinical management of nasopharyngeal carcinoma patients undergoing radiotherapy. Malnutrition can diminish sensitivity to radiochemotherapy, exacerbate side effects, reduce treatment tolerance, and impair patient quality of life (13–15).

With growing awareness of the nutritional challenges faced by patients undergoing radiotherapy for nasopharyngeal carcinoma, various nutritional support approaches are now accessible. Early nutritional intervention (16–18) is recognized as beneficial for enhancing patients’ nutritional status. Nutritional education combined with oral nutritional supplementation (ONS) has been shown to enhance patients’ nutritional intake (19–23), improve tolerance to radiotherapy (24), and decrease the occurrence of adverse reactions to radiotherapy in nasopharyngeal cancer patients through enteral nutrition (25, 26). In terms of nutritional intervention, there are a variety of intervention strategies available, such as personalized whole nutritional management (27–33), systematic nutritional management (34), the plan-do-check-act (PDCA) cycle model (35), intensive management mode (36, 37), and the multidisciplinary collaboration model (38, 39). These intervention methods are believed to enhance the nutritional status of nasopharyngeal cancer patients undergoing radiotherapy and improve their tolerance to the treatment. However, these intervention and management strategies have developed from other diseases and are built upon the framework of nutritional management for oncology patients. Yet, they lack the specificity and targeted guidance for nutritional support tailored to patients with nasopharyngeal carcinoma undergoing radiotherapy.

So far, there is only an expert consensus on nutritional support for nasopharyngeal cancer patients, with recommendations limited to nutritional risk screening and assessment, nutritional education, selection of nutritional treatment modalities, and nutrient requirements (7). Notably, there is a lack of guidance on nutritional monitoring and follow-up. Additionally, specific guidelines for treating nasopharyngeal carcinoma patients undergoing radiotherapy are absent, with simplified content devoid of specific recommendations. Consequently, nurses find it challenging to utilize this tool for guiding clinical practice. In this study, we systematically conducted a literature search on studies related to nutritional support for patients undergoing nasopharyngeal carcinoma radiotherapy. We then screened and integrated the available evidence with the aim of providing the best clinical practices for nutritional support in this patient population.

## 2 Methodology

### 2.1 Establishment of evidence-based questions

The PIPOST model, developed by the JBI Center for Evidence-Based Nursing at Fudan University in Shanghai, served as the framework for constructing evidence-based queries. P (population) identifies the specific target population: patients with nasopharyngeal carcinoma undergoing radiotherapy; I (intervention) denotes the nutritional support intervention; the second P (professional) refers to the professionals applying the evidence: clinical medical staff; O (outcome) focuses on the incidence of malnutrition and patients’ nutritional status; S (setting) specifies the location where the evidence is applied: radiology wards; T (type of evidence) outlines the variety of evidence types utilized, including guidelines, systematic evaluations, expert consensus, best clinical practice information booklets, evidence summaries, and original research.

### 2.2 Evidence retrieval

This study searched the following databases and websites:

The following Chinese databases were used: China National Knowledge Infrastructure (CNKI), Wanfang Database, VIP Full Text Database, and China Biology Medicine (CBM).

The following English databases were used: PubMed, Embase, Web of Science, Cochrane Library, and CINAHL.

Guidelines networks: the Scottish Intercollegiate Guidelines Network (SIGN), BMJ Best Practice, National Guideline Clearinghouse (NGC), Ding Xiangyuan, Medlive.

Relevant nutrition society websites include the official websites of the European Society for Parenteral and Enteral Nutrition, the American Society for Parenteral and Enteral Nutrition, the American Academy of Nutrition and Dietetics (AND), and the Chinese Society for Nutrition.

Search strategy. The search terms included “Nasopharyngeal Carcinoma,” “Carcinoma, Nasopharyngeal,” “Head and Neck Neoplasms,” “Radiotherapy,” “Radiation Treatment,” “Targeted Radiotherapy,” “Nutritional Status,” “Nutrition Disorders,” “Diet, Food, and Nutrition,” “Enteral Nutrition,” and “Parenteral Nutrition,” with a search period from 2013 to 2023.

### 2.3 Inclusion and exclusion criteria

The inclusion criteria for the present study were patients who underwent radiotherapy for nasopharyngeal carcinoma; nutritional support for patients who underwent radiotherapy for nasopharyngeal carcinoma; guidelines, expert consensuses, summaries of evidence, systematic evaluations, and original research; and written in Chinese and English.

The exclusion criteria were as follows: guideline interpretations and plans; studies repeatedly published or updated; studies with incomplete information or unavailable full text; and studies with failed quality evaluation.

### 2.4 Literature screening

The literature was screened independently by two postgraduate students in the group who had received training in evidence-based nursing, and the screening steps were as follows: ① deduplication: Endnote software was used to de-adjust duplicates; ② initial screening: the titles and abstracts of the literature were read, and the literature that was not relevant to the topic was excluded; ③ rescreening: the remaining literature was read carefully, the eligible literature was screened, and the basic information of the literature was extracted. The screening results of the two researchers were cross-checked, and when the results were controversial, a third evidence-based care specialist was invited to determine the inclusion status.

### 2.5 Evaluation of the quality of the literature

For evaluating guidelines, we employed the Clinical Guidelines Research and Evaluation System (AGREE II), which assesses various aspects, including “scope and purpose,” “participants,” “rigor of development,” “clarity,” “applicability,” and “editorial independence.” This tool consists of 23 individual entries and two overall evaluation entries. Each entry is scored on a scale of 1–7, with higher scores indicating better compliance. The standardized percentage score for each item is calculated using the formula: (actual score – minimum possible score)/(maximum possible score – minimum possible score) × 100%. Items with standardized percentage scores ≥60% are categorized as Grade A, those with scores ≥30% but <60% as Grade B, and items with scores <30% as Grade C.

Systematic evaluations, meta-analyses, expert consensus, randomized controlled trials, cohort studies, and observational studies underwent assessment using the Australian JBI Centre for Evidence-Based Health Care’s Quality Assessment Criteria (2016) (40). Evaluators rendered judgments of “yes,” “no,” “unclear,” or “inapplicable” for each item based on the literature. Following group discussion, decisions were reached regarding inclusion, exclusion, or the need for further information for each item labeled as “no,” “unclear,” or “not applicable.”

The quality of the included studies was assessed separately by two researchers who had undergone evidence-based training. In cases of disagreement, the judges were assisted by deliberation or by a third evidence-based care specialist.

### 2.6 Evidence extraction and summary

Two researchers with evidence-based training and more than a decade of clinical experience extracted and summarized evidence from the included literature. The grading of evidence followed the Australian Center for Evidence-Based Health Care’s Level of Evidence Recommendation System (2014 version). Any disagreements were resolved through consultation or with the aid of a third researcher. In instances of conflicting evidence conclusions, priority was given to high-quality and recently published evidence from peer-reviewed journals.

## 3 Results

### 3.1 Search results

**Figure 1:**
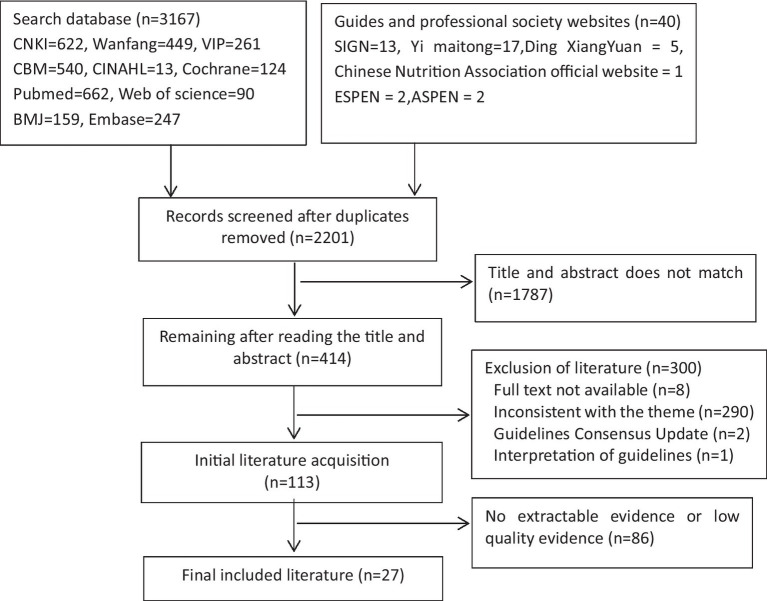
A flow chart of literature screening.

Initially, 3,207 articles were gathered, and after deduplication using ENDNOTE, 2,201 articles remained. Xiaomei Fan and Shasha Liu, both trained in evidence-based medicine, independently screened the titles and abstracts of these articles. They excluded 1,787 articles deemed irrelevant, leaving 414 articles for further review. Following a full-text assessment, eight articles were excluded due to unavailability, 290 for being off-topic, 2 for being updated guidelines, and 1 for being an interpretation of guidelines. This left 113 articles for careful scrutiny. Upon examination, 86 articles were further excluded due to either the inability to extract evidence or low evidence quality, resulting in the selection of 27 articles. These included six guidelines, eight expert consensuses, four systematic evaluations, five randomized controlled trials, two quasi-experiments, and two observational studies. The process of literature screening is outlined in Figure 1.

The general characteristics of the included studies are shown in Table 1.

**Table 1:** The general characteristics of the included literature.

Title of literature	Type of literature	Literature sources	Year of publication
ESPEN practical guideline: clinical nutrition in cancer	Guideline	Clinical Nutrition	2021
SEOM clinical guidelines on nutrition in cancer patients (2018)	Guideline	Clin Transl Oncol	2019
Oncology evidence-based nutrition practice guideline for adults	Guideline	Journal of the Academy of Nutrition and Dietetics	2017
Nutritional management in head and neck cancer: United Kingdom national multidisciplinary guidelines	Guideline	The Journal of laryngology and otology	2016
Chinese guidelines for radiotherapy of nasopharyngeal cancer (2022 edition)	Guideline	Chin J Cancer Prev Treat	2022
Guidelines on nutritional support in patients with tumor	Guideline	Chinese Journal of Surgery	2017
Consensus and clinical recommendations for nutritional intervention for head and neck cancer patients undergoing chemoradiotherapy in Taiwan	Expert consensus	Oral Oncology	2018
Expert opinion on nutritional treatment for patients with nasopharyngeal carcinoma	Expert consensus	Electron J Metab Nutr Cancer	2021
Dietary nutrition prescription for cancer patients	expert consensus	Electron J Metab Nutr Cancer	2017
Expert consensus on nutrition therapy for radiotherapy patients	Expert consensus	Electron J Metab Nutr Cancer	2021
Expert consensus on standardized management of radiotherapy nutrition	Expert consensus	Chin J Radiat Oncol	2020
Expert consensus on nutrition support therapy for head and neck cancer patients receiving radiotherapy	Expert consensus	Chin J Radiat Oncol	2018
Oral nutrition supplements consensus for cancer patients undergoing radiotherapy (2017)	Expert consensus	Chin J Radiat Oncol	2017
Expert consensus on appetite assessment and regulation in cancer patients	Expert consensus	Electron J Metab Nutr Cancer	2020
Effect of nutritional interventions on nutritional status, quality of life and mortality in patients with head and neck cancer receiving (chemo) radiotherapy: a systematic review	Systematic review	Clinical nutrition	2013
Effect of oral nutritional supplements with or without nutritional counselling on mortality, treatment tolerance, and quality of life in head and neck cancer patients receiving (chemo)radiotherapy: a systematic review and meta-analysis cancer patients receiving (chemo)radiotherapy: a systematic review and meta-analysis	Systematic review	The British Journal of Nutrition	2021
Consumption of processed food and risk of nasopharyngeal carcinoma: a systematic review and meta-analysis	Systematic review	Translational Cancer Research	2022
Relationship of diet and lifestyle with the risk of nasopharyngeal carcinoma among Chinese population: a meta-analysis	Systematic review	J Prev Med Inf	2020
A prospective randomized controlled trial on the value of prophylactic oral nutritional supplementation in locally advanced nasopharyngeal carcinoma patients receiving chemo-radiotherapy	Randomised controlled experiments	Oral oncology	2020
Benefits of oral nutritional supplements in patients with locally advanced nasopharyngeal cancer during concurrent chemoradiotherapy: an exploratory prospective randomized trial	Randomised controlled experiments	Nutrition and cancer	2018
Effect of oral supplements on the nutritional status of nasopharyngeal carcinoma patients undergoing concurrent chemotherapy: A randomized controlled Phase II trial. Controlled Phase II trial	Randomised controlled experiments	Journal of cancer research and therapeutics	2020
Effect of modified yangyin qingfei decoction combined with nutritional intervention on nutritional status and curative effect of nasopharyngeal carcinoma patients undergoing radiotherapy	Randomised controlled experiments	Cancer Res Prev Treat	2019
Effect of oral nutritional supplements on nutritional status and quality of life in patients with nasopharyngeal carcinoma receiving chemoradiotherapy	Randomised controlled experiments	China Oncology	2018
Impact of parenteral glutamine supplement on oncologic outcomes in patients with nasopharyngeal cancer treated with concurrent chemoradiotherapy	quasi-experiment	Nutrients	2022
Effect of early nutrition intervention on advanced nasopharyngeal carcinoma patients receiving chemoradiotherapy	Quasi-experiment	J Cancer	2019
Prognostic value of nutritional risk screening 2002 scale in nasopharyngeal carcinoma: a large-scale cohort study	Observational research	International Journal of Radiation Oncology Biology Physics	2018
The value of two nutritional screening tools in the nutritional assessment of patients undergoing radiotherapy for nasopharyngeal carcinoma and their correlation with cancer-caused fatigue	Observational research	Chinese nursing Research	2021

### 3.2 Results of the evaluation of the quality of the included studies

#### 3.2.1 Results of the evaluation of the quality of the guidelines

Six guidelines were included in the study, with their quality assessment results presented in Table 2. Two of these guidelines originated from China (5), The Chinese Society of Parenteral and Enteral Nutrition (45), offering recommendations for radiation therapy in nasopharyngeal carcinoma patients and nutrition management in cancer patients, respectively. One guideline was from the United States (43), focusing on nutrition support guidelines for cancer patients. Another guideline was from the UK, providing evidence-based nutritional practice guidelines for adults with cancer (44). Additionally, one guideline was from Spain (42), titled the SEOM clinical guidelines for cancer patient nutrition (2018), and one was from Europe, focusing on clinical nutrition guidelines (41). Except for Kang Min’s guideline (5), which specifically addressed nasopharyngeal carcinoma radiotherapy, the remaining guidelines pertained to nutrition. Among them, three articles received Grade A recommendations, while three received Grade B recommendations.

**Table 2:** Guide quality evaluation results.

Guideline	Percentage standardization by area (%)						≥60%	30–60%	Recommended level
	①	②	③	④	⑤	⑥			
Muscaritoli et al. (41)	94.44	94.44	97.91	94.44	95.83	91.67	6	0	A
De Las et al. (42)	83.33	77.78	56.25	77.78	54.17	91.67	5	1	B
Thompson et al. (43)	94.44	83.33	66.67	77.78	83.33	75.00	6	0	A
Talwar et al. (44)	66.67	44.44	35.42	77.78	45.83	16.67	2	3	B
Kang (5)	94.44	77.78	43.75	94.44	81.81	91.67	5	1	B
Chinese Society for Parenteral and Enteral Nutrition (45)	88.89	77.78	85.41	94.44	91.67	66.67	6	0	A

① Scope and purpose; ② stakeholder involvement; ③ rigour of development; ④clarity and presentation; and ⑤ applicability; ⑥Editorial independence.

#### 3.2.2 Quality evaluation results of the expert consensus

Nine expert consensuses from China were included in the study. These were authored by Lin et al. (46), Li (7), Li et al. (47), Li et al. (13), Branch of Radiation Oncology of Chinese Medical Association (48), Cancer Radiotherapy Nutrition Group, Cancer Nutrition and Support Committee of China, China Anti-Cancer Association (49), China Society for Radiation Oncology (50), and Li et al. (51). Seven were written in Chinese, while one was in English. Among them, four articles discussed nutritional therapy (7, 13, 46, 48), one focused on ONS (50), one addressed diet nutrition (47), and one explored appetite regulation in cancer patients (51). The expert consensus was included in this study, and there was a high inter-rater agreement. All of the entries were yes, except for entry 6, “Is there any inconsistency between the proposed viewpoints and the previous literature?” The evaluation for each criterion is outlined, as detailed in Table 3.

**Table 3:** Results of the evaluation of the quality of expert consensus.

Expert consensus	①	②	③	④	⑤	⑥
Lin et al. (46)	Yes	Yes	Yes	Yes	Yes	No
Li (7)	Yes	Yes	Yes	Yes	Yes	No
Li et al. (47)	Yes	Yes	Yes	Yes	Yes	No
Li et al. (13)	Yes	Yes	Yes	Yes	Yes	No
Chinese Medical Association Radiation Oncology Therapy Branch (48)	Yes	Yes	Yes	Yes	Yes	No
Cancer Radiotherapy Nutrition Group, Cancer Nutrition and Support Committee of China, China Anti-cancer Association (49)	Yes	Yes	Yes	Yes	Yes	No
China Society for Radiation Oncology (50)	Yes	Yes	Yes	Yes	Yes	No
Li et al. (51)	Yes	Yes	Yes	Yes	Yes	No

① Is the source of the opinion clearly identified? ② does the source of the viewpoint have standing in the field of expertise? ③ Are the interests of relevant groups the central focus of opinions? ④ Is the stated position the result of the analysis process? Is the viewpoint expressed logically? ⑤ Do you have any references to existing literature? and ⑥ Whether there is any inconsistency between the viewpoints presented and the previous literature.

#### 3.2.3 Systematic evaluation or meta-analysis quality evaluation results

Three systematic evaluations and one meta-analysis were included in the study: Langius et al. (52), Mello et al. (53), Feng et al. (54), and Chen et al. (55). Feng et al. (54) investigated the impact of processed foods on nasopharyngeal cancer patients, while Mello et al. (53) and Langius et al. (52) explored the effects of nutritional interventions on patients undergoing head and neck radiotherapy. Chen et al. (55) examined the correlation between lifestyle and dietary behaviors in a Chinese nasopharyngeal cancer population. The evaluation results can be found in Table 4.

**Table 4:** Systematic evaluation or meta-analysis evaluation results.

Inclusion of literature	①	②	③	④	⑤	⑥	⑦	⑧	⑨	⑩	⑪
Langius et al. (52)	Yes	Yes	Yes	Yes	Yes	Yes	Yes	Yes	Yes	Yes	Yes
Mello et al. (53)	Yes	Yes	Yes	Yes	Yes	Yes	Yes	Yes	Yes	Yes	Yes
Feng et al. (54)	Yes	Yes	Yes	Yes	Yes	Yes	Yes	Yes	Yes	Yes	Yes
Chen et al. (55)	Yes	Yes	Yes	Yes	Yes	Yes	Yes	Yes	Yes	Yes	Yes

① Did the research questions and inclusion criteria for the review include the components of PICO? ② whether the literature inclusion criteria were appropriate for that evidence-based question; ③ whether the search strategy used was appropriate; ④ whether the sources of the research papers were appropriate; ⑤ whether the criteria used to evaluate the quality of the literature were appropriate; ⑥ whether the evaluation of the quality of the literature was done independently by two or more evaluators; ⑦ whether the data were extracted with measures to reduce errors; ⑧ whether the methods used to synthesize/combine studies were appropriate; ⑨ whether possible publication bias was assessed; ⑩ whether recommendations for policy and/or practice were made with the support of the reported data; and ⑪ whether appropriate recommendations were made for future directions for further research.

#### 3.2.4 Results of the quality assessment of randomized controlled trials

Five randomized controlled trials were incorporated into the study: Huang et al. (24), Jiang et al. (23), Dou et al. (19), Huang et al. (51), and Ding et al. (15). Huang et al. (51) examined the utilization of traditional Chinese medicine decoctions in nutritional intervention for nasopharyngeal carcinoma patients. The remaining four articles explored oral nutritional supplementation in nasopharyngeal carcinoma patients. The evaluation results are provided in Table 5.

**Table 5:** Results of quality evaluation of randomized controlled trials.

Inclusion of literature	①	②	③	④	⑤	⑥	⑦	⑧	⑨	⑩	⑪	⑫	⑬
Huang et al. (24)	Yes	Yes	Yes	No	Unclear	Unclear	Yes	Unclear	Yes	Yes	Yes	Yes	Yes
Jiang et al. (23)	Yes	Yes	Yes	No	No	No	Yes	Yes	yes	Yes	Yes	Yes	Yes
Dou et al. (19)	Unclear	Unclear	Yes	Unclear	Unclear	Unclear	Yes	No	Yes	Yes	Yes	Yes	Yes
Huang et al. (56)	Yes	Unclear	Yes	Unclear	Unclear	Unclear	Yes	Yes	Yes	Yes	Yes	Yes	Yes
Ding et al. (21)	Yes	Unclear	Yes	Unclear	Unclear	Unclear	Yes	Yes	Yes	Yes	Yes	Yes	Yes

① Whether to truly use random grouping; ② whether to achieve hidden allocation; ③ is the baseline between groups comparable; ④ whether blinding was applied to the research subjects; ⑤ whether to implement blinding for the interveners; ⑥ whether blinding is applied to the evaluators of the results; ⑦ are the other measures received by each group the same, except for the intervention measures to be verified; ⑧ whether the follow-up is complete and whether measures have been taken to deal with lost follow-up; ⑨ will all randomly assigned research subjects be included in the analysis of the results; ⑩ whether to use the same method to evaluate the outcome indicators of each group of research subjects; ⑪ is the evaluation method for outcome indicators appropriate; ⑫ is the data analysis method appropriate; ⑬ and whether the research design is reasonable.

#### 3.2.5 Results of the evaluation of the quality of quasi-experiments

Two quasi-experimental studies were included. Wang et al. (57) investigated the effects of parenteral glutamine supplementation on nasopharyngeal carcinoma patients undergoing concurrent radiotherapy, while Meng et al. (16) explored the effects of early nutritional intervention on nasopharyngeal cancer patients. Evaluation results are detailed in Table 6.

**Table 6:** Quality evaluation results of quasi-experiments.

Inclusion of literature	①	②	③	④	⑤	⑥	⑦	⑧	⑨
Wang et al. (57)	Yes	Yes	Yes	Yes	Yes	Yes	Yes	Yes	Yes
Meng et al. (16)	Yes	Yes	Yes	Yes	Yes	Yes	Yes	Yes	Yes

① Has the causal relationship in the study been clearly explained; ② is the baseline between groups comparable; ③ are the other measures received by each group the same, except for the intervention measures to be verified; ④ has a control group been established; ⑤ whether to implement diversified measurement of outcome indicators before and after the intervention; ⑥ is the follow-up complete? If incomplete, have you reported the loss of follow-up and taken measures to address the issue; ⑦ whether to use the same method to evaluate the outcome indicators of each group of research subjects; ⑧ is the evaluation method for outcome indicators reliable; ⑨ and is the data analysis method appropriate.

#### 3.2.6 Quality evaluation results of observational studies

Two observational studies were included. A study by Peng et al. (58) demonstrated the use of the NRS-2002 in the nutritional screening of nasopharyngeal cancer patients, and a study by Luo et al. (59) compared the value of the two nutritional screening tools in the nutritional assessment of nasopharyngeal cancer radiotherapy patients. The evaluation results are shown in Table 7.

**Table 7:** Results of quality evaluation of observational studies.

Inclusion of literature	①	②	③	④	⑤	⑥	⑦	⑧
Peng (58)	Yes	Yes	Yes	Yes	Yes	Yes	Yes	Yes
Luo et al. (59)	Yes	Yes	Yes	Yes	Yes	Yes	Yes	Yes

① Whether the inclusion criteria for the sample were clearly defined; ② whether the study population and site were described in detail; ③ whether the exposure factors were measured in a valid and reliable manner; ④ whether the results were measured using objective evaluation criteria; ⑤ whether confounding factors were identified; ⑥ whether measures were taken to address the confounding factors; ⑦ whether the method of outcome measurement was credible; and ⑧ whether the method of data analysis was appropriate.

### 3.3 Summary and description of evidence

We summarized the 60 pieces of best available evidence in 6 areas, namely, nutritional risk screening and assessment, nutrient requirements, nutritional support, nutritional symptom management, functional exercise, and nutritional monitoring and follow-up. The details are shown in Table 8.

**Table 8:** Results of literature extraction and grading of evidence.

Level 1 entries	Secondary entries	Tertiary entries	Level
Nutritional risk screening and assessment	The time of Screening and assessment	1. Nutritional assessment and comprehensive measurements should be routinely performed in patients with nasopharyngeal carcinoma after admission (7, 49). 2. Nasopharyngeal cancer patients should receive NRS2002 risk screening at least once a week during their treatment, in order to detect the nutritional risk of nasopharyngeal cancer patients as early as possible and provide early nutritional intervention (7, 49).	5b 5b
	Screening and assessment tools	1. The NRS 2002 scale is recommended for nutritional risk screening (7, 13, 45, 47–50, 58). 2. The PG-SGA scale is recommended for nutritional assessment if there was a risk (7, 13, 44–49, 59).	3d 5b
	Content of the assessment	1. For patients with abnormal screening, an objective and quantitative assessment of nutritional intake, symptoms of nutritional effects, muscle mass, physical performance and degree of systemic inflammation are performed (41, 42, 45).	5b
Nutrient requirement	Energy	1. The recommended energy requirement for patients with nasopharyngeal carcinoma is 25–30 kcal/ (kg-d) (7, 13, 41, 42, 45, 47–50). 2. If the patient has a combination of severe complications, energy requirements are recommended to be 30–35 kcal/ (kg-d) (7).	5b 5b
	Carbohydrate	1. Whole grains, starchy vegetables are recommended as a source of carbohydrates (47).	5b
	Protein	1. The amount of protein supplied should be 1.0 to 1.5 g∕kg per day, and fish, meat, eggs and milk are recommended as sources of protein (7, 41, 47, 49).	5b
	Lipid	1. Nutritional therapy for patients with nasopharyngeal carcinoma should appropriately increase the proportion of fat for energy supply, and fat intake generally does not exceed 30% of total energy (7, 45, 47, 49).	5b
	Water	1. It is recommended to consume 30–40 mL/kg of water per day. If vomiting or diarrhea occurs, additional supplementation is required (47).	5b
	Micronutrient	1. Supplement the physiological requirements of vitamins and trace elements to avoid deficiency of vitamins and trace elements in the body (41, 45, 47).	5b
	Pharmacological nutrients	1. Supplementation with enteral nutrition preparations rich in- ω3 polyunsaturated fatty acids (PUFA) may be useful in reducing the inflammatory response and maintaining the patient’s weight (13, 45, 46). 2. Glutamine supplementation is recommended to reduce chemotherapy- and radiotherapy-induced mucositis in patients with nasopharyngeal carcinoma (13, 45, 46, 57). 3. The addition of probiotics regulates the balance of the host’s intestinal bacterial flora and improves the patient’s metabolic and immune status (47). 4. Add antioxidant nutrients including vitamin C, carotenoids, lycopene, and so on to reduce the side effects of radiation and chemotherapy (47).	5b 2d 5b 5b
Nutritional support	Timing and principles of nutritional support	1. Routine nutritional support is not recommended for radiotherapy patients in good nutritional status, and early nutritional intervention is recommended for patients at nutritional risk or malnutrition (16, 41, 44, 45, 48, 50). 2. Nutritional therapy should follow the five-step principle: ① diet and nutritional education; ② oral nutritional supplementation; ③ enteral nutrition EN; ④ partial enteral PEN and partial parenteral PPN; and ⑤ total parenteral nutrition TPN (7, 13, 45, 48, 50).	2d 5b
	Diet + nutrition education	1. Nasopharyngeal cancer patients should receive regular dietary and nutritional education during the perioperative period of radiation therapy (5, 45, 48, 50–52). 2. Adequate nutrient intake is achieved through dietary guidance and/or oral nutritional supplementation (41, 42, 50). 3. Cooking in a healthy way, with steaming, boiling, braising and stir-frying as the mainstay, less frying, deep-frying and roasting, and reducing the amount of fat, salt, and soy sauce and monosodium glutamate (47). 4. Tumor patients can increase their food intake by increasing the number of meals as appropriate, eating smaller meals or eating whenever they feel hungry (47). 5. Eat less pickled vegetables, cured meats and processed foods that increase the incidence of nasopharyngeal cancer, quit smoking and limit alcohol, drink more milk, and eat fresh vegetables and fruits (47, 54, 55).	5b 5b 5b 5b 2b
	Oral nutritional supplementation (ONS)	1. When the body’s nutritional needs are not met by improved oral intake because of intensive nutritional education, the ONS is needed (15, 23, 24, 42, 45). Routine prophylactic placement of nutritional tubes prior to radiation therapy is not recommended (13, 46, 48). 2. If symptom control is unsatisfactory or inadequate dietary intake has been resolved, but the patient continues to lose weight, the dietitian may consider the use of a specialty medical food containing eicosapentaenoic acid (EPA) (43). 3. Nutritional preparations for radiotherapy oncology patients should be in holistic formulations (50).	1c 5b 5b
	Enteral nutrition (EN)	1. If oral food intake is inadequate despite nutritional education and ONS, EN should be used (41, 45). 2. Enteral nutrition should also be initiated if inadequate food intake (60% of estimated energy expenditure) is expected for more than 10 days (44). 3. In nasopharyngeal cancer patients with difficulty in feeding through the mouth, a trans nasal feeding tube should be chosen for short-term enteral nutrition. And percutaneous endoscopic gastrostomy (PEG) or percutaneous endoscopic jejunostomy (PEJ) is required if the expected feeding time is >4 weeks (5, 7, 13, 41, 42, 44, 45, 49). 4. Prophylactic placement of a nutritional tube may be considered in patients with one or more of the following conditions: significant weight loss (>5% at 1 month or > 10% at 6 months), BMI <18.5 kg/m2, severe dysphagia or pain, severe anorexia, and anticipation of severe radiation oral or esophageal mucositis (13, 44).	5b 5b 5b 5b
	Parenteral nutrition (PN)	1. Nasopharyngeal cancer patients with gastrointestinal dysfunction should be treated with parenteral nutrition or combined parenteral + enteral (5). 2. Parenteral nutrition is used if adequate oral/enteric nutrition cannot be provided (42). 3. Patients who experience severe adverse reactions (e.g., severe mucositis), inability to eat normally, or significant reduction in food intake during radiotherapy should receive parenteral nutrition (7, 42, 45, 49, 50).	5b 5b 5b
	Chinese medicine	1. Chinese medicine can improve patients’ appetite and quality of life by enhancing immunity, regulating the spleen and stomach, and inhibiting cytokines (5, 45, 48, 50, 52). 2. The combination of nutritional intervention with Jiawei Nourishing Yin and Clearing Lung Soup can improve the nutritional status and biochemical indexes of patients and ensure the progress and efficacy of radiotherapy (56).	5b 2c
Nutritional symptom management	Oral mucositis	1. Choose foods that are soft and tender in texture, easy to chew, and finely ground, cooking it by steaming (51). 2. If you have heartburn after meals, try to stay in a standing or sitting position for 1 h after the meals (51). 3. Maintain oral and nasal hygiene, gargle frequently, remove food residues in the mouth and teeth, or use mouthwash prescribed by the doctor to promote wound healing (5, 51). 4. A straw can be used for fluid intake to minimize food contact with the wound (51). 5. Patients should stop smoking and limit alcohol and avoid stimulating diets (47, 51). 6. If you have a candida infection, treat it promptly (5).	5b 5b 5b 5b 5b 5b
	Xerostomia	1. You can eat sweeter or more acidic food to increase the secretion of saliva, but patients with pain and swelling of the mouth should be cautious (51). 2. Food choices or cooking styles: eating soft or finely chopped foods to make them easier to swallow or mixing them into soups; changing cooking styles, e.g., thickening, steaming, making soups (51). 3. Eat small mouthfuls of food and chew thoroughly. Consume a sufficient amount of water daily and drink in small sips to moisturize the oral mucosa. If the symptom of xerostomia is severe and painful swelling occurs, medications to protect the oral mucosa can be used as prescribed by the doctor (51).	5b 5b 5b
	Dysgeusia	1. Depending on the patient’s preferences, try to choose foods that the patient feels colorful and flavorful (51). 2. Use seasonings appropriately to change the flavor of food (51).	5b 5b
	Anorexia	1. Glucocorticoids or progestins may be applied to improve anorexia in patients with tumors for short periods of time, but possible adverse effects must be considered (41, 45, 51). 2. Fish, chicken, eggs, soy products and milk can be used to instead of pork and beef, but should be careful to avoid fishy smell (51).	5b 5b
functional exercise	Head and neck functional exercises	1. Pay attention to strengthening mouth opening exercises and neck rotation exercises during radiotherapy (5). 2. Patients insist on swallowing function training to maintain and improve swallowing function (46).	5b 5b
	Muscle exercise	1. Patients are advised to maintain or increase their level of physical activity to support muscle mass, body function, and metabolic patterns (41, 42). 2. It is recommended that you start with a small amount of exercise, 5 to 10 min a day, and gradually reach 150 min of exercise (e.g., walking) per week according to your physical condition. Generally speaking, the best state of exercise is to sweat slightly all over the body and not feel tired (47).	5b 5b
Nutritional monitoring and follow-up	Nutritional monitoring	1. After implementing nutritional interventions, patients should be evaluated regularly for nutrition (47). 2. Physical Examination Indicators: weight, percent change in weight, body mass index, grip-strength (44, 47). 3. Laboratory indices: serum albumin, prealbumin, electrolytes, C-reactive protein (44, 47). 4. Nutritional intake (44).	5b 5b 5b 5b
	Follow up	1. After radiotherapy, professional nutritionists or medical staff will conduct regular nutritional follow-up and monitoring of patients, and provide nutritional support or home nutrition treatment if necessary (48). 2. Nutritional follow-up every 2–4 weeks, lasting for 3 months or until radiotherapy-induced chronic adverse effects, weight loss, or nasogastric tubes are properly resolved (44, 48). 3. NRS2002 Nutritional Risk Screening is performed monthly for 3 months after radiotherapy (48). 4. Follow-up includes weight, dietary intake, intake of nutritional supplements and modalities, and the presence of intolerance symptoms (48). 5. Follow-up includes telephone follow-up, text message reminders, home visits, and regular discharge education (48).	5b 5b 5b 5b 5b

Nutritional risk screening and assessment involve three secondary entries and five tertiary entries. Both the consensus on nutritional support for nasopharyngeal carcinoma and the consensus on nutrition and supportive care for patients undergoing radiotherapy for head and neck tumors (7, 49) advocate for routine nutritional risk screening and assessment upon admission. The NRS2002 is recommended as a nutritional risk screening tool, while the PG-SGA is endorsed as an assessment tool. This recommendation is supported by multiple expert consensuses (7, 13, 47, 50) and guidelines (45). Furthermore, a large-scale cohort study (58) also suggested the use of NRS2002 as a nutritional risk screening tool for nasopharyngeal carcinoma patients. Patients with abnormalities should undergo timely, comprehensive assessments and early nutritional interventions (7, 49).

Nutrient requirements include seven secondary entries for energy, carbohydrates, proteins, fats, water, trace elements, and pharmacological nutrients, along with eleven tertiary entries. Nutrients serve as essential raw materials for sustaining organisms and are vital for ensuring proper nutrition. Several guidelines (41, 42, 45) and consensus documents (7, 13, 47–50) concur that the recommended energy intake for nasopharyngeal carcinoma patients ranges from 25 to 30 kcal/(kg·d). In cases of severe complications, the recommended energy intake increases to 30 to 35 kcal/(kg/d) (7). Additionally, recommendations for nutrient intake have been provided. Li et al. (47) outlined specific recommendations regarding food sources and cooking methods for proteins, carbohydrates, and other nutrients. Nutraceuticals such as glutamine and probiotics are also considered effective; Wang’s (57) findings suggest that parenteral supplementation of glutamine can mitigate side effects, thereby recommending it as a nutraceutical.

Nutritional support consists of six secondary entries and nineteen tertiary entries, covering topics like the timing and principles of nutritional support, diet and nutritional education, oral nutritional supplementation, enteral nutrition, parenteral nutrition, and traditional Chinese medicine. Patients undergoing radiation therapy for nasopharyngeal carcinoma should receive timely nutritional support if they are at risk of malnutrition (16, 41, 44, 45, 48, 50). An intervention study (16) demonstrated the beneficial effects of early nutritional intervention in maintaining patients’ nutritional status and improving treatment tolerance. Nutritional support follows a five-step treatment principle (7, 13, 45, 48, 50), including diet and nutritional education, oral nutritional supplements, enteral nutrition, and parenteral nutrition, as recommended by various guidelines and consensus documents. Diet and nutritional education are ongoing aspects of the nutritional support process, with regular provision of diet + nutritional education (5, 45, 48, 50–52). Oral intake is the simplest and most economical method. Emphasizing healthy cooking methods (47) and consuming small, frequent meals (47) is recommended for oral intake. Expert consensus (47) and two meta-analyses (54, 55) also suggest minimizing the consumption of pickled vegetables, cured meats, and processed foods. Oral nutritional supplements (ONS) are employed (23, 24, 42) when dietary intake and nutritional education are insufficient, supported by clinical evidence and guideline recommendations (42, 45). If ONS fails to meet nutritional needs, enteral nutrition (EN) is initiated, and if EN is inadequate, parenteral nutrition (PN) is considered. Additionally, in some studies, traditional Chinese medicine, an integral part of China’s medical heritage, has shown supportive effects in nutritional support for cancer patients (56).

Management of nutritional symptoms comprises four secondary symptoms: oral mucositis, dry mouth, taste disorders, and anorexia, along with twelve tertiary entries. Side effects induced by radiotherapy often disrupt patients’ nutritional intake, yet appropriate food choices and cooking methods can alleviate the impact of these symptoms. The expert consensus by Li et al. (51) offers detailed recommendations for preventing and treating nutritional symptoms.

Functional exercises include two secondary entries, head and neck functional exercises, and muscle exercises, along with four tertiary entries. Due to the potential stiffness of head and neck muscles induced by radiotherapy, patients are advised to incorporate exercises to strengthen neck-turning, mouth-opening (5), and swallowing (46). Furthermore, expert consensus (47) and guidelines (41, 42) suggest that encouraging patients to participate in suitable activities can improve bodily tolerance and facilitate nutrient absorption.

Nutritional monitoring and follow-up consist of two secondary entries and nine tertiary entries. Post-implementation of nutritional interventions, both guidelines (44) and consensus (47) advocate for regular monitoring of outcomes, including physical examination parameters, laboratory test results, and nutritional intake. Upon patient discharge, expert consensus (48) suggests follow-up by professional nutritionists or medical personnel every 2–4 weeks to assess the patient’s nutritional status. This can be achieved through various means, such as telephone follow-up, WeChat correspondence, or home visits.

## 4 Discussion

Malnutrition is a prevalent issue among patients undergoing radiotherapy for nasopharyngeal carcinoma and is linked to adverse outcomes (54). Currently, there is a shortage of evidence-based strategies for providing nutritional support to these patients. To improve nutritional care for individuals undergoing radiotherapy for nasopharyngeal carcinoma, this study conducted an extensive literature review, evaluating the quality of available literature and summarizing evidence on six aspects of nutritional support programs: nutritional risk screening and assessment, nutrient requirements, nutritional support, nutritional symptom management, functional exercise, and nutritional monitoring and follow-up.

### 4.1 Emphasizing the importance of nutritional risk screening and assessment and early identification of nutritional risks

Patients diagnosed with nasopharyngeal carcinoma are at significant risk of malnutrition, with some already malnourished at the time of diagnosis. As treatment progresses, their nutritional status tends to worsen. Hence, early initiation of nutritional risk screening is crucial. Regular screenings not only detect high-risk individuals early but also enable timely nutritional support, reducing intolerance occurrences. This protocol outlines the timing, tools, and content for nutritional risk screening and assessment. Following guideline recommendations, we selected the NRS2002 (7, 13, 45, 47, 48, 50, 58, 60) for nutritional risk screening, scoring based on impaired nutritional status, disease severity, and age. For nutritional assessment, the PG-SGA is recommended (7, 13, 44–50, 59), comprising both patient-generated subjective global assessment and healthcare professionals’ evaluation (61). A meta-analysis comparing seven nutritional screening tools in cancer patients (62) revealed the PG-SGA’s superior sensitivity and predictive value, making it suitable for newly diagnosed cancer patients’ nutritional screening. Studies (63) have confirmed the NRS2002’s applicability for nutritional risk screening and the PG-SGA for nutritional assessment in nasopharyngeal carcinoma patients. While the NRS2002 and PG-SGA are widely used, other tools like MNA, MUST (64), and GLIM (12) can also serve for nutritional risk screening and assessment. As nutritional concerns gain prominence, body composition analyzers (65), CT, and metabolic carts are being employed for nutritional assessment, albeit at added costs. CT and MRI involve radiation, and operating metabolic carts could be complex. The NRS2002 and PG-SGA offer simplicity, high specificity, patient safety, and no added economic burden, making them suitable choices based on patients’ clinical circumstances.

### 4.2 Reasonably adding nutrients to ensure nutrient requirements

Adequate nutritional substances are essential for patients undergoing radiotherapy for nasopharyngeal carcinoma, forming the foundation of nutritional support. This plan outlines the patient’s nutritional requirements and provides corresponding substance recommendations, including energy, carbohydrates, proteins, fats, water, trace nutrients, and pharmacologic nutrients. Proteins play a crucial role as the fundamental building blocks of life. Research (66) suggests that a diet rich in amino acids can delay the onset of sarcopenia in tumor patients undergoing radiotherapy and chemotherapy. Plant-derived fats, particularly unsaturated fatty acids, are preferred over saturated fatty acids. Two prospective cohort studies conducted in the United States (67) have demonstrated that substituting animal-sourced polyunsaturated fatty acids with plant-derived alternatives can reduce mortality rates. The addition of micronutrients such as glutamine, probiotics, and antioxidants is recommended to mitigate radiotherapy side effects. Studies indicate that parenteral glutamine supplementation can enhance energy intake in head and neck cancer patients (68) and decrease side effect incidence (40, 57, 69, 70). Probiotics have emerged as crucial players in tumor-patient immune regulation. A randomized controlled study indicated (71) that probiotics could lower Candida infections in head and neck cancer patients undergoing radiotherapy. Additionally, probiotics may reduce oral mucositis incidence by modulating immunity and gut microbiota (72–74). It has been indicated that probiotics and gut microbiota will likely become integral components of cancer prevention and treatment in the coming years (75). However, it is important to note that guidelines typically do not recommend routine pharmacologic nutrient supplementation. Clinical supplementation may be considered based on individual patient conditions.

### 4.3 Reasonable nutritional support to improve nutritional status

The nutrition support section includes eight aspects: timing of nutritional support, principles of nutritional support, diet and nutrition education, ONS, TEN, PEN and PPN, TPN, and traditional Chinese medicine (TCM). Currently, our country’s focus on nutritional support is inadequate, leading to irregular applications of EN and PN. A nutritional support survey study involving 526 hospitalized cancer patients (76) revealed that among 245 patients scoring ≥3 on the NRS2002, the nutritional support rate was only 59.6%. Among them, 131 patients received PN, while only 15 patients were provided with EN, resulting in a parenteral to enteral nutrition ratio of 8.7:1. Hence, there is a pressing need to prioritize and standardize nutritional support. Early nutritional support should be promptly offered to patients at nutritional risk, while those without such risk may not require routine support. However, early support can help sustain the patient’s nutritional status and minimize side effects (16). Research also indicates that preemptive ONS can enhance tolerance to radiotherapy and chemotherapy (19). Therefore, whether to administer routine nutritional support to patients undergoing radiotherapy for nasopharyngeal carcinoma should be assessed based on individual circumstances and preferences. Tumor nutritional therapy adheres to the principle of frontline treatment, prioritizing diet, oral nutrition, nutrition education, and enteral nutrition, guided by a five-tier treatment approach (77). Additionally, TCM plays an active role in cancer adjuvant therapy (78), aiding in weight maintenance during radiotherapy (79), delaying oral mucositis onset (80), and enhancing quality of life (81). Studies (56) demonstrate that integrating Yangyin Qingfei Decoction with nutritional interventions can enhance patients’ nutritional status and biochemical indicators. Future endeavors should further advance TCM intervention research to explore the synergistic effects of TCM and Western medicine on cancer patients’ nutritional status.

### 4.4 Emphasize symptom management

In symptom management, guidelines and consensus offer recommendations for addressing conditions like oral mucositis, dry mouth, taste disturbances, and anorexia. Patients are advised to select suitable foods and employ appropriate cooking methods to alleviate symptoms. However, caution is advised when considering the use of glucocorticoids or progestogens to enhance appetite, given potential adverse reactions. Oral mucositis, closely linked to the patient’s nutritional status (8), has spurred considerable research into alleviation strategies. Honey (82–84), thalidomide (85), probiotics (72–74), and oral glutamine (70) have demonstrated efficacy in reducing oral mucositis. Taste disturbances, commonly reported post-radiation therapy, significantly impact patients’ quality of life (86). While options like radiation therapy mode, taste field dose distribution, and occlusal blocks may prove effective, current evidence remains inconclusive, necessitating further investigation.

### 4.5 Strengthening functional exercise

As a result of radiotherapy’s adverse effects, patients often face challenges such as limited mouth opening and swallowing difficulties post-treatment. Hence, engaging in functional exercises becomes crucial. Alongside exercises targeting mouth and neck mobility to maintain oral and pharyngeal function, it is vital to gradually introduce whole-body muscle training to enhance overall physical health and resilience against illnesses. Studies suggest that multimodal exercise regimens can improve the physical well-being of nasopharyngeal cancer patients undergoing radiotherapy (87). Additionally, practices like the Eight-Section Brocade have shown promise in enhancing patients’ quality of life (88), while Tai Chi has demonstrated efficacy in alleviating tumor-related fatigue (89).

### 4.6 Ensure proper nutritional monitoring and follow-up

Following nutritional support, it is essential to conduct regular nutritional assessments, including physical examination indicators, laboratory parameters, and dietary intake. Based on the evaluation outcomes, adjustments to the nutritional support regimen can be made promptly. Upon discharge, dedicated professionals should conduct regular follow-ups with the patient. A customized follow-up strategy can be devised to address the patient’s specific nutritional concerns promptly and offer appropriate guidance.

## 5 Conclusion

This study aimed to consolidate the most reliable evidence on nutritional support for patients undergoing radiotherapy for nasopharyngeal carcinoma, intending to furnish a comprehensive blueprint for clinical nutritional intervention. However, since factors like hospital resources, patient preferences, financial capabilities, insurance coverage, and cultural beliefs can influence the implementation of such schemes, future endeavors should incorporate real-world clinical insights. Consulting expert opinions will be pivotal in refining nutritional support strategies for these patients. Furthermore, clinical validation of these interventions is imperative to enhance patient nutrition, improve treatment endurance, and diminish side effects.

### 5.1 Limitations of the study

Although this study provides evidence of nutritional support for patients with nasopharyngeal carcinoma undergoing radiotherapy, factors such as regional, ethnic, and cultural differences may affect the results. In addition, this study only searched for the literature in Chinese and English. Future updates should continuously update the data, explore the applicability and feasibility of the evidence, be combined with clinical updates, and promote the application of evidence in the clinic.

## Data availability statement

The original contributions presented in the study are included in the article/supplementary material, further inquiries can be directed to the corresponding author.

## Author contributions

XF: Conceptualization, Investigation, Methodology, Resources, Writing – original draft, Writing – review & editing. HC: Conceptualization, Funding acquisition, Methodology, Supervision, Validation, Writing – original draft, Writing – review & editing. SL: Data curation, Formal analysis, Supervision, Validation, Writing – review & editing.
